# Clinical Efficacy Evaluation of Tuina Combined Medicated Oil in the Treatment of Nonspecific Low Back Pain: Protocol for a Randomized Controlled Trial

**DOI:** 10.2196/81887

**Published:** 2026-03-11

**Authors:** Man Yuan, Guangxin Guo, Siyu Wang, Fei Yao, Guimao Wang, Hongxi Xu

**Affiliations:** 1 School of Pharmacy, Shanghai University of Traditional Chinese Medicine, 1200 Cailun Road, Pudong, Shanghai, 201203, China, 86 021-51323089, 86 021-51323089; 2 Shanghai Municipal Hospital of Traditional Chinese Medicine, Shanghai University of Traditional Chinese Medicine, Nunmber 274 Zhijiang Middle Road, Jing'an District, Shanghai, 200071, China

**Keywords:** Tuina, medicated oil, non-specific low back pain, clinical effect, randomized controlled trial

## Abstract

**Background:**

Nonspecific low back pain (NSLBP) is a significant global public health concern that affects the health and well-being of individuals across different age groups, limiting their daily activities and reducing their quality of life. As Tuina (Chinese therapeutic massage) therapy and medicated oil are widely used in China, it is necessary to design a randomized clinical trial to assess the effectiveness of Tuina combined medicated oil (TNO) in treating NSLBP.

**Objective:**

This study aims to evaluate the efficacy and safety of Tuina therapy combined with medicated oil in treating NSLBP.

**Methods:**

One hundred participants will be enrolled and randomly allocated to either a TNO group (n=50) or a Tuina combined water group (n=50). Treatment will last for 4 weeks, with sessions 3 times a week, followed by a 4-week follow-up. The visual analog scale score is the primary outcome; secondary outcomes include the evaluation of treatment Japanese Orthopaedic Association scores, infrared thermography, muscle tension tests, and tenderness scores. All adverse reactions will be recorded.

**Results:**

The trial commenced in June 2023 and is expected to conclude in September 2025. In June 2025, key preliminary steps were completed, and the ethical review and clinical trial registration were concluded. Recruitment is proceeding as planned, with 100 participants enrolled to date. Data collection is underway, while formal data analysis has not yet commenced.

**Conclusions:**

The project’s purpose is to evaluate the effectiveness of TNO in alleviating pain and improving lumbar function in patients with NSLBP.

## Introduction

Nonspecific low back pain (NSLBP) clinical symptoms include low back pain, muscle tension, stiffness below the rib margin and above the gluteal sulcus, leg pain, numbness, and weakness. NSLBP is one of the leading causes of disability among workers in high-income and transitional countries, ranking sixth in overall disease burden [1,2]. NSLBP is a significant global public health issue that impacts individuals, their health, and society. It often recurs and imposes a burden on the economy. It is the most expensive medical condition in the United States, estimated at US $134.5 billion in 2016 [3,4].

According to an epidemiological survey [5], the incidence rate of NSLBP increased by 18% from 2006 to 2016. With the change of people’s lives and learning styles, low back pain is increasingly affecting younger individuals. A study of 404,206 adolescents from 28 countries found that 37% experienced low back pain every month or more often [6]. Risk factors associated with NSLBP include obesity, lack of exercise, poor occupational or lifestyle habits, depression, and other social and psychological factors [7]. Maintaining a normal weight, good physical health, proper posture, and regular exercise may reduce the risk, but evidence-based support is lacking [8,9].

NSLBP accounts for the highest proportion of low back pain [1]. NSLBP affects individuals of different age groups, limits their daily activity function, and reduces their quality of life [10,11]. There is no specific treatment for NSLBP; the purpose of therapy is to alleviate pain and its consequences, including any disabilities related to NSLBP. Traditional Chinese medicine (TCM) external treatment methods, Tuina, offer unique advantages in clinical applications, as they are noninvasive, have relatively low treatment costs, are simple to apply, and are highly acceptable [12-14]. Clinical studies on treating lower back pain with Tuina, both domestically and internationally, have demonstrated that massage therapy effectively reduces muscle and myofascial tension, promoting local metabolism [12,13,15,16].

Currently, the treatment methods for patients with NSLBP are divided into surgical and nonsurgical therapies. Nonsurgical therapies include cognitive behavioral therapy, exercise, manual therapy, physical therapy, acupuncture, and pharmacological therapy [17-20]. The North American Spine Society indicates that there is currently insufficient evidence to recommend or oppose a fusion surgery for lower back pain. There is also insufficient evidence to suggest that imaging results of lumbar fusion are associated with better clinical outcomes, with a level I recommendation [14]. Different treatment methods have their advantages and disadvantages. Surgical therapy has high costs, while traumatic treatment is insufficient, and its traumatic nature limits its effectiveness. Nonsurgical therapies, such as exercise therapy, can be challenging to adhere to. The practical and convenient treatment methods must be explored in clinical practice. Tuina acts on the body’s surface through mechanical stimulation as an alternative and complementary therapy. Patients easily accept Tuina therapy and feel highly comfortable. Medicated oil is an effective, safe, and easy-to-use treatment [21,22]. However, clinical trials of Tuina combined medicated oil (TNO) for treating lower back pain generally have a low level of evidence-based methods, lack a high-quality randomized controlled trial design, and are not included in clinical diagnosis and treatment guidelines.

Tuina has unique advantages as one of the traditional external treatment methods in TCM. Previous studies have confirmed its role in various waist-related diseases such as NSLBP, lumbar disc herniation, and acute lumbar sprain [23-26]. Tuina therapy emphasizes the balance of muscles and bones, prioritizing tendons and the presence of tendons that can lead to dislocation of bones, both of which are primary pathogenic factors. Tuina has a holistic view of TCM in the practice of “principles, methods, formulas, and techniques,” and has the characteristics of benign and bidirectional regulation in the treatment mechanism. It has been widely applied in clinical practice to treat musculoskeletal diseases.

The Flying Eagle Wood Lok Medicated Oil (FEMO) used in this study has effects that include warming meridians, dispelling cold, wind, and dampness, promoting blood circulation, and relieving pain. Medicated oil is a traditional Chinese patent medicine and a simple preparation made according to the principles of TCM. It exhibits characteristics of percutaneous penetration, rapid onset, and minimal gastrointestinal side effects.

The main components of FEMO consist of *Panax notoginseng* (Burk.) F.H. Chen, *Aconitum carmichaelii* Debx., *Arisaema erubescens* (Wall.) Schott, *Pinellia ternata* (Thunb.) Breit, *Carthamus tinctorius* L., methyl salicylate (wintergreen oil), menthol, camphor, eucalyptus oil, lavender oil, rose oil, and olive oil [27,28]. Modern medical research has shown that medicated oil has anti-inflammatory, analgesic, and preventive effects on pressure ulcers [29]. It is applied to treat muscle soreness, rheumatic bone pain, old and new sprains, arthritis pain, mosquito bites, sprain swelling and pain, low back and leg tendon pain, bone spurs, sciatica, and other diseases. NSLBP is one of its indications. For usage, follow the instructions and apply Tuina techniques, including finger pressure, for 15‐20 minutes to enhance the effectiveness of the drugs.

Although Tuina is widely used in the prevention and treatment of NSLBP, the current literature on its efficacy often lacks a rigorous experimental design, significantly weakening the persuasiveness of the research results. Although clinical trials have demonstrated effectiveness, no relevant evidence exists to explain the mechanism of action, which also applies to traditional Chinese patent medicines and simple preparations when rubbed with Tuina. This study is based on combining manual therapy with the external application of medicated oil in treating NSLBP, to fully leverage the complementary advantages of manual therapy combined with external treatment drugs. This study aims to provide an effective method for treating NSLBP and to objectively evaluate the clinical application of medicated oil by combining TCM Tuina with medicated oil.

## Methods

### Ethical Considerations

This study has been approved by the Ethics Committee of Shanghai Municipal Hospital of Traditional Chinese Medicine (2023SHL-KY-96-01). All participants provided written informed consent before participation. Participant privacy and confidentiality were strictly protected throughout this study; all data were anonymized and stored securely, and no personally identifiable information was disclosed. Participants received no compensation.

### Study Design

**Figure 1.**
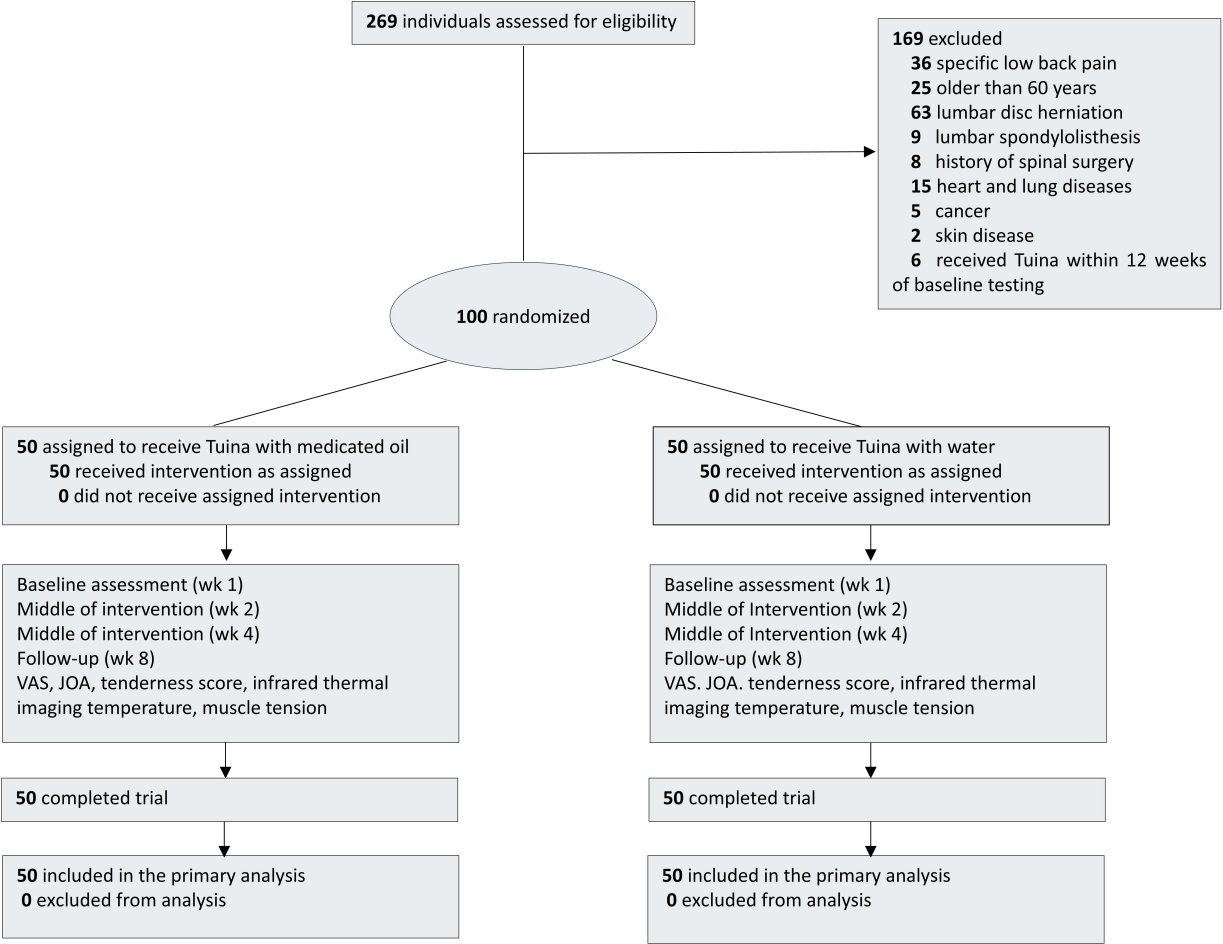
The trial flowchart. This study is a randomized controlled trial. A total of 100 patients will be randomly divided into 2 groups, the TNO group and the TNW group. Each group has the same number of patients, and the trial will include a 4-week treatment period. During the treatment process, patients in the TNO group received massage combined with medicated oil intervention, while patients in the TNW group received massage combined with water intervention. Two outcome evaluations will be conducted at 5 time points: baseline, the first week, the second week, the fourth week of treatment, and the eighth week of follow-up. JOA: Japanese Orthopaedic Association scores; TNO: Tuina combined medicated oil; TNW: Tuina combined water; VAS: visual analog scale.

**Figure 2.**
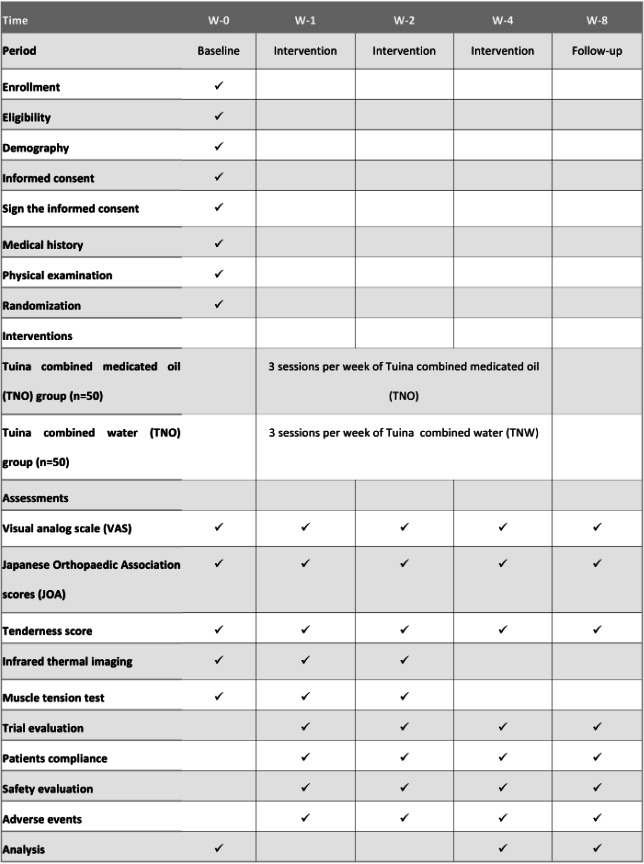
This study’s schedule shows the time points (wk [W]) for enrollment and assessment.

The research is a single-site study, parallel-controlled, randomized controlled trial. We will recruit 100 patients with NSLBP from the Shanghai Municipal Hospital of Traditional Chinese Medicine. Participants will receive informed consent during the recruitment process and will have equal opportunities to be randomly allocated to the TNO or Tuina combined water (TNW) groups. Due to limitations in intervention methods, outcome evaluators and statisticians will be kept blinded to the treatment assignment. Evaluators will evaluate and analyze the results at 5 time points (preintervention, 1, 2, and 4 wk after intervention, and 1-mo follow-up). Data management and statistical analysis will be performed at the Shanghai TCM Hospital Research Department. Figure 1 provides a visual representation of the research design, while Figure 2 displays the research schedule.

### Sample Size Calculation

Based on the preliminary experimental research results, the sample size was estimated, and G*Power (version 3.1; Heinrich-Heine-Universität Düsseldorf) software was used to calculate the sample size for each group. The sample size was calculated to detect a between-group difference in the change in visual analog scale (VAS) score from baseline to week 4 (end of treatment), based on pilot posttreatment data. No power calculations were performed for week 8 outcomes, which are considered exploratory.

For grasp 1, the testing level was set at *α*=.05 and *β*=.95, and the required sample size was calculated to be 42 per group, considering a 10% dropout rate. According to 1:1 parallel grouping, VAS’s mean for the 2 pre-experimental groups was 3.57 (SD 1.35) in the TNO group and 2.61 (SD 1.39) in the TNW group. Using the above parameters, 50 patients with NSLBP should be included in each of the 2 groups, yielding a mean difference of 0.96 and a pooled SD of 1.37 (corresponding to a Cohen *d* effect size of 0.70), indicating a moderate-to-large treatment effect. The high statistical power was chosen to ensure robust internal validity, particularly given the modest, single-center study design and potential overestimation of the effect size from pilot data.

### Participants Recruitment

This study used a randomized controlled, single-blind trial method, with participants recruited from the outpatient departments of Shanghai Municipal Hospital of TCM. A total of 100 patients were randomly assigned to an observation group (TNO) and a control group (TNM), with 50 patients in each group (Textbox 1).

Textbox 1. **Inclusion and exclusion criteria.**

Inclusion criteria

- Meeting the diagnostic criteria for the nonspecific low back pain set by the North American Spine Society and Chinese expert consensus [30,31].
- Being between the ages of 20 and 59 years.
- During the trial period of the research method, immediately stopping any therapies that may affect the determination of research effectiveness.
- Participating in this study, cooperating with the doctor’s arrangements, and signing an informed consent form.

Exclusion criteria

- Excluding specific low back pain lesions such as lumbar disc herniation and lumbar spondylolisthesis, and those related to bone joints and the spinal canal.
- Being patients with severe heart, lung, liver, and kidney diseases.
- Being individuals undergoing other clinical trials.
- Being individuals with a history of spinal surgery, spinal injury or deformity, or fracture.
- Being patients with combined acute infections, cancer, rheumatic and immune diseases, and mental illnesses.
- Being individuals with other primary functional disorders.

### Dropout Criteria

The exclusion criteria were (1) not persisting in treatment or not following the treatment plan, (2) voluntarily requesting withdrawal, (3) having other serious concurrent diseases during the treatment process, and (4) using other treatment methods in combination.

### Washout Period and Concomitant Treatments

No formal washout period was required before enrollment. To minimize potential confounding, all concomitant treatments used before enrollment, including physical therapy and medications, were comprehensively documented at baseline. During this trial period, participants were required to discontinue any additional therapies that could interfere with outcome assessment immediately, as specified in the inclusion criteria.

### Randomization and Allocation Concealment

Using SPSS (version 27.0; IBM Corp) for Windows software, a random number table was generated and randomly grouped into 2 sets in order of enrollment: the observation group, treated with a combination of Tuina and medicated oil; and the control group received the same Tuina technique, but the activating oil was changed to water, forming a control group with the treatment group.

### Blinding

Due to the nature of manual therapy combined with topical application, complete blinding of all parties is inherently challenging. Several measures were implemented to maximize blinding feasibility. They were the following: (1) participants neither handled nor observed the bottles during treatment, and allocation will be concealed from all the participants; (2) the patients will be informed that they will receive one of two effective interventions, which will be randomly assigned after enrollment; (3) this study’s coordinator will notify participants of the randomized results and schedule any necessary interventions; (4) the group allocation will be kept concealed from data managers, statisticians, and evaluators throughout the procedure to minimize bias; (5) medicated oil and water are packed in bottles of the same shape, size, and color, and the bottles are encoded according to a random sequence; and (6) the bottle cap of water is soaked in activating oil to replicate its odor. In practical Tuina applications, apart from its odor, the medicated oil used in this study exhibits physical properties comparable to those of water, including fluidity, spreadability, viscosity, and skin contact characteristics. The Tuina practitioner and the cutaneous sensation experienced by participants during manipulation are broadly similar between the 2 intervention media in handling properties, which reduces the likelihood that therapists can reliably distinguish group allocation during treatment based on touch alone, thereby further limiting potential operator-expectation bias.

### Observation Group (TNO Group)

The dedicated attending physician of the Tuina Department is responsible for the treatment of Tuina. Tuina doctors have graduated from the Tuina College of Shanghai University of TCM, majoring in acupuncture, moxibustion, and Tuina, and have been occupied with clinical Tuina work for 5‐8 years. We finished standardized training on the techniques before treatment begins and ensured patients are treated only after passing the assessment. According to the diagnostic and treatment standards, the Tuina techniques established by the expert group will be used for intervention. The specific plan is as follows:

1. Rubbing tendons is applied to the paraspinal erector spinae muscle of the lumbar spine using the palm root kneading method. Up and down reciprocating operation is performed in 3 minutes. Apply, in 3 minutes, the thumb Tuina method to the attachment area of the iliac crest muscle, the affected buttocks, popliteal fossa, and calf, focusing on the areas where the patient feels sore and swollen.
2. Apply the thumb folding finger flicking technique to the lumbar quadratus muscle, transverse process of the third lumbar vertebra, and Ashi acupoint (tenderness point or local cord-like or nodular area), with 3‐5 flicks per area, reaching the affected area. To loosen adhesions and relieve spasms for 2 minutes.
3. Grab the affected buttocks and calf muscles for 2 minutes.
4. The patient is advised to take a standing position and perform extreme movements such as waist flexion, lateral flexion to the left and right, and forward flexion to the left and right for 2 minutes. This is to stretch the muscles in the lower back.
5. The patient is prone when using medicated oil for external rubbing. According to the instructions, take 8‐10 drops of the medicated oil. First, apply the medicated oil evenly to the affected area using the palm-rubbing method, and then take 6‐8 drops of the medicated oil. Use the palm rubbing method to horizontally rub the lumbar and sacral areas and vertically rub the Du meridian and foot sun meridian tendons, each about 5‐7 times, with a degree of heat transmission. At the pain point, use techniques combined with activating meridian oil to perform Tuina and pluck for 8 minutes.

The treatment frequency is 3 times a week, for 20 minutes each time.

**Figure 3.**
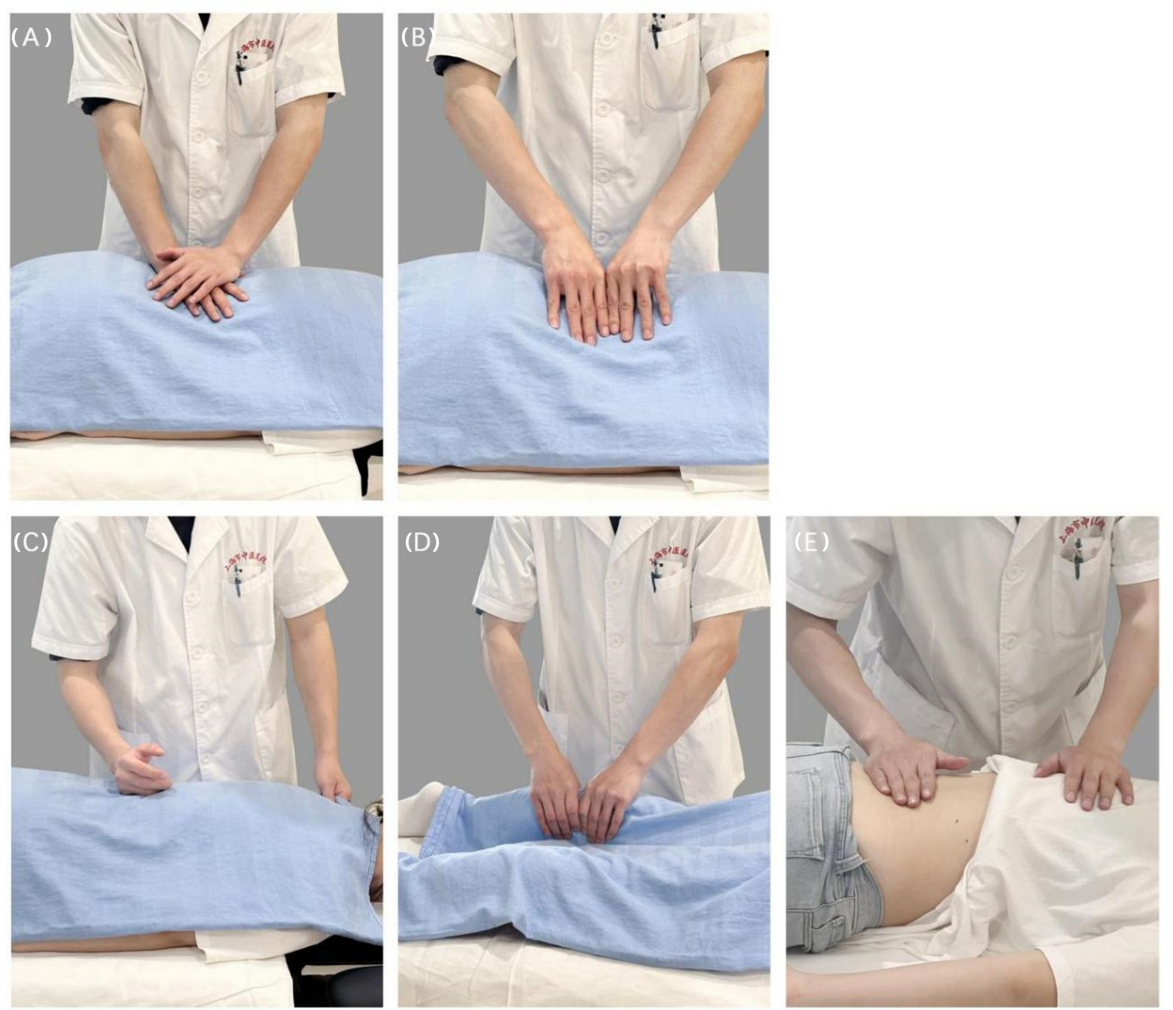
(A) Lumbar flexion, (B) left lumbar flexion, (C) right lumbar flexion, (D) left anterior lumbar flexion, and (E) right anterior lumbar flexion.

The treatment cycle is for 4 weeks, with a total of 12 treatments. The specific action characteristics are shown in Figures 3A-3E.

### Medicated Oil Drug Information

The brand was FEMO (executive standard number: JZ2017004).

The product registration number was ZC20170003, with a specification of 50 mL.

The medicinal ingredients were *P notoginseng* (Burk) F.H. Chen, *A carmichaelii* Debx., *A erubescens* (Wall.) Schott, *P ternata* (Thunb.) Breit, *C tinctorius* L., methyl salicylate (wintergreen oil), menthol, camphor, eucalyptus oil, lavender oil, rose oil, and olive oil.

FEMO is a government-approved proprietary Chinese medicine manufactured under good manufacturing practice standards. The alkaloids from *A carmichaelii* are strictly controlled during processing to ensure compliance with safety requirements. The product is approved for external use and has been lawfully marketed in Hong Kong (registration number: HKC-00369).

### Control Group (TNW Group)

Using the Tuina technique in the above plan, we performed step 5. When we applied the medicated oil externally, we replaced the medicated oil with clean water to form a control.

The treatment frequency was 3 times a week, for 20 minutes each time. The treatment cycle was 4 weeks.

### Outcome Measurement

Through the combination of VAS, Japanese Orthopaedic Association scores (JOA), tenderness score, infrared thermal imaging, and muscle tension testing, this study comprehensively evaluates the effectiveness of the intervention, gathering both subjective (patient-reported) and objective (physiological) outcomes. The primary end point is the change in VAS score from baseline to week 4 (end of treatment). VAS is assessed at baseline and weeks 1, 2, 4, and 8; however, the week 4 assessment serves as the prespecified primary analysis time point.

### Primary Outcome

The VAS scoring standard, also known as the pain level scoring system, is a visual analog scale method used to evaluate the severity of pain. A 10-cm-long straight line is used, with both ends representing 0 points for painless and 10 points for extreme pain. The scale is: 0 is no pain, 1‐3 indicates mild pain, 4‐6 moderate pain, and 7‐10 severe pain. After introducing the key points, the patient will self-evaluate the degree of pain based on their feelings [32].

### Secondary Outcome Measurements

#### About JOA

The JOA scale evaluates both subjective symptoms and objective signs. The total score of the scale is 29 points, assessed across 5 aspects and 25 items [33]. The score is directly proportional to the function. The larger the scale score, the milder the subjects’ symptoms.

#### Infrared Thermal Imaging

Using infrared thermal imaging (FLIR ONE Pro, FLIR Systems, Inc) measurement method: maintain indoor temperature at 26 ℃±2 ℃, humidity at 45%-60%, maintain stable indoor air without convection, and avoid direct wind blowing to the examination site or between the subject and the camera. The subject shall not take any medication that affects body temperature orally within 24 hours, shall not undergo physical therapy, and shall not drink alcohol. The subject should lie prone, rest for a moment, maintain a certain balance in body temperature, wait for the skin to dry and become sweat-free, and then expose the tested area for 5 minutes before taking photos. The corresponding colors on the infrared image from high temperature to low temperature are white, deep red, red, light red, yellow, green, light green, light blue, deep blue, and black. Identify the pain points in the patient’s area and mark them accordingly. Measure the temperature of the pain points in the pain area before enrollment, at week 1, at week 2, and after week 4 of treatment. The location of the pain points in the pain area is determined based on palpation, patient feedback, and the first infrared thermography image. A low temperature in the pain area before treatment indicates poor local blood circulation and a decreased pain threshold. A high temperature at pain points after treatment indicates good local blood circulation and an increased pain threshold. The testing unit we used was ℃.

#### Muscle Tension Test

We use a muscle hardness tester (OE-220 from Ito Ultra Short Wave Co, Ltd)×1 unit. Our test method is that the subject lies prone, fully exposing the skin on the waist and back, and naturally relaxes their hands, placing them on both sides of the body. Mark 8 points, which are 1.5 and 3 cun to the left and right of the L2 spinous process, and 1.5 and 3 cun left and right of the L4 spinous process. Press vertically and slowly on the surface of the measured muscle to prompt the patient to stop pressing. The obtained data will be displayed on the display screen. If the data are correct, press the record button to record the data. After 3 measurements, the display screen directly shows the average test value. The range of values is 0‐99.99, where a small value represents high muscle fiber elasticity and low muscle tension. In contrast, a significant value represents low muscle fiber elasticity and high muscle tension, expressed as a percentage of the total muscle tension. Of measurement frequency, the two groups were measured before enrollment, at week 1, at week 2, and after treatment at week 4.

#### Tenderness Score

A normal score of 0 is defined as no significant pain during heavy pressure or maximum activity.

A mild score of 1 point is defined as when under heavy pressure, the patient reports pain, but passive activity is not restricted as such.

A moderate score of 2 is defined as when under heavy pressure, the patient reports pain and frowns, indicating discomfort, with mild restricted mobility.

A severe score of 3 points is defined as when under heavy pressure, the patient reports pain and withdrawal, and passive activity is severely restricted.

For measurement frequency, the 2 groups were measured before enrollment, at week 1, at week 2, at week 4, and after treatment at week 8.

#### Adherence

At the start of this study, a group of individuals with an interest in Tuina treatment of NSLBP will be evaluated to assess their suitability for participation in the experiment. The assessment will involve collecting their medical history, conducting specialized examinations, and administering auxiliary tests under the supervision of doctors. Participants can choose whether to participate after fully understanding the grouping and procedures. Researchers will complete standardized training courses and regularly review the course videos to monitor the accuracy of the intervention measures. Before and after each treatment, a dedicated research assistant is responsible for evaluating and recording data, which is recorded in the case report form (CRF). Any exceptional circumstances that arise during treatment should be promptly reported to the Tuina doctor and documented in the CRF.

#### Safety Evaluation

The category and severity of adverse events (AEs) after each intervention are recorded in detail on the CRF, including drug allergies, subcutaneous bleeding, joint injuries, hypertension, headache, dizziness, tinnitus, fever, and other relevant conditions. The incidence of AEs will be reported as the proportion of AEs to the total number of treatments. If the AE does not improve after sufficient rest, participants should receive timely medical treatment. Suppose a serious AE occurs, the chief investigator and the ethics committee will be well-informed within 24 hours of the event. They will be knowledgeable for additional assessment and treatment and decide whether to terminate the trial. Researchers also continue to monitor patients who have experienced AE until the issue is settled, especially individuals who withdrew due to AE. If AE is proven to be related to the intervention measures in this study, participants will receive appropriate medical and economic compensation.

The medicated oil used in this study contains ingredients with known warming or irritant properties (methyl salicylate, menthol, and processed *Aconitum* species). A multilevel monitoring strategy has been implemented to ensure participant safety. First, FEMO is a government-approved proprietary Chinese medicine manufactured under good manufacturing practice standards, with strict control of potentially toxic constituents, and is approved exclusively for external use.

During each treatment session, participants are actively observed by trained Tuina physicians for any signs of abnormal local skin reactions (excessive erythema, burning sensation, pain, or discomfort at the application site). The observations are systematically documented using tenderness scores, muscle tension tests, and structured patient feedback and are collected at predefined time points.

Infrared thermal imaging is used as an objective tool to monitor local skin temperature changes. Moderate temperature elevation is considered a physiological response reflecting improved microcirculation; disproportionate or excessive temperature increases may indicate abnormal inflammatory or irritant responses.

All AEs, particularly skin-related reactions, are recorded in detail after each intervention. Participants are instructed to report any discomfort immediately. If adverse reactions occur, appropriate medical evaluation and intervention are initiated, and events are followed until resolution. Serious AEs are reported to the principal investigator and ethics committee within 24 hours, in accordance with the predefined safety management protocol.

#### Quality Control

Quality control will be conducted under the guidance of an independent oversight committee to eradicate potential biases and guarantee the quality of the experiment. The steering committee comprises investigators with expertise in Tuina trials, statistical design, medication, and AEs. They are accountable for monitoring data, identifying issues, reviewing collected data, and controlling biases. The protocol will remain unchanged during the 8-week treatment period, to ensure consistency in delivery.

All physicians and evaluators are required to complete standard training. Additionally, experienced Tuina physicians will be required to complete standard training courses with nationwide recognized academic experts who have 5‐8 years of clinical experience in Tuina before the trial commences. The treatment plan should strictly follow the protocol of Tuina treatment steps, including the selection of Tuina techniques, intensity, time, sequence, and NSLBP protection measures.

Randomly assign to 2 groups simultaneously to avoid differences in the degree of NSLBP due to treatment physician and allocation bias. Participants need to cooperate in completing the entire treatment plan and follow-up.

#### Data Collecting and Monitoring

Clinical trial data will be obtained through CRFs, paper questionnaires, or online data collection applications and stored securely. Confidentiality will be maintained by assigning an encrypted numerical code to each individual. The accuracy of data input has been double-checked. The data will be locked and analyzed by 2 statisticians under the supervision of a third statistician and administrator. Then, record this trial’s data on the subwebsite of the Chinese Clinical Trial Center [34], the electronic data management system. After the data input is completed, the electronic database will be closed. Management personnel must report weekly to the steering committee on the progress of data monitoring, including the accuracy and reliability of the data. Experimental audits encompass regular, independent reviews of core experimental processes and documents, including data and scheduling monitoring, to ensure the integrity of experimental results. Data monitoring is the responsibility of the Research Department at Shanghai Municipal Hospital of TCM. The supervisor will verify that all AEs are recorded in the correct format and that they are consistent with the protocol definition. Monitoring personnel will verify the following variables for all patients: name abbreviation, date of birth, gender, signed informed consent form, eligibility criteria, randomization date, treatment allocation, AEs, and end point. The arrangement of monitoring visits will depend on the patient’s registration status, the site’s status, and other commitments. Investigators must meet with monitors at all times. If problems are discovered during the visit (ie, insufficient personnel for research and lack of research documents), the supervisor will assist in resolving the problem on-site. We will access electronic monitoring to review source files and confirm AEs.

#### Statistical Analysis

Although the sample size calculation was based on a simplified between-group comparison at the primary postintervention time point (wk 4), the primary outcome analysis will use a longitudinal modeling framework to account for repeated measurements appropriately. A linear mixed-effects model will be used, including fixed effects for treatment group, time, and group-by-time interaction, with a random intercept specified for each participant. An unstructured covariance matrix will be prespecified to model within-subject correlations across repeated VAS measurements (baseline, and wk 1, 2, 4, and 8). Sensitivity analyses using alternative covariance structures, including first-order autoregressive and compound symmetry matrices, will be conducted to assess the robustness of the results. The data analysis was performed using SPSS (version 27.0) statistical software, and the testing criteria were as follows: *α*=.05, with *P*<.05 considered statistically significant.

Given the longitudinal design with repeated measurements collected at 5 time points (baseline, and wk 1, 2, 4, and 8), all primary and secondary continuous outcomes will be analyzed rather than with independent *t* tests or simple ANOVA, which accounts for within-subject correlation and avoids violations of the independence and sphericity assumptions inherent in traditional methods.

In each linear mixed-effects model, fixed effects will include treatment group (TNO vs TNW), time (modeled as a categorical variable), and the group-by-time interaction, with a random intercept specified for each participant. An unstructured covariance matrix will be prespecified for the repeated measures, with sensitivity analyses using alternative covariance structures (autoregressive and compound symmetry) to assess robustness. The primary estimand is the between-group difference in change from baseline to week 4 (end of treatment), which is defined a priori as the primary time point based on the sample size calculation.

To control multiplicity, a hierarchical testing strategy will be applied. The primary end point (change in VAS from baseline to wk 4) will be tested at a 2-sided α level of .05. Secondary outcomes (JOA score, tenderness score, muscle tension, and infrared thermography) will be tested sequentially only if the preceding end point reaches statistical significance, thereby controlling the family-wise error rate. Longitudinal group-by-time interactions across all postbaseline time points will be considered exploratory.

Clinically meaningful improvement will be evaluated using responder analyses in addition to continuous outcomes. A reduction of ≥2 points on the VAS and an improvement of ≥4 points on the JOA score will be defined a priori as the minimal clinically significant difference. The proportions of responders will be compared between groups using generalized estimating equations, with effect sizes reported as risk ratios and 95% CIs.

#### Analysis of the Primary Outcome

The primary outcomes will be evaluated at baseline, intervention 1 week, 2 weeks, 4 weeks, and follow-up. We will conduct a sensitivity analysis on missing data (intention-to-treat analysis). The primary end point is the average change in the VAS scale from baseline to week 4 (primary end point) and week 8 (durability assessment). The baseline covariates include height, gender, age, cardiovascular history, and weight. The entire outcome scale will be measured at baseline. When the distribution is normal, nonpaired *t* tests are used to compare 2 groups, and 1-way ANOVA and repeated measures ANOVA are used to compare multiple groups. Sensitivity analysis using multiple input techniques (chain equation inputs) will be included to evaluate the impact of missing data on the main results. In addition, we will compare participants randomly assigned to the TNO and TNW groups, who received treatment considered to have high fidelity allocation. The percentage of missing data is 20%, and 5 input datasets will be used for analysis, which is the recommended minimum quantity. Before conducting any analysis, investigate any missing data patterns, identify the reasons for the missing data, and investigate the patterns to determine the validity of the hypothesis.

#### Analysis of Secondary Outcomes

Refer to the primary outcome analysis and conduct a secondary analysis of the outcomes. We will test the interarm differences for each specified end point. AE analysis was performed using the chi-square or Fisher exact test.

## Results

This trial commenced in June 2023 and is expected to conclude in September 2025. As of June 2025, the initial steps have been completed. Specifically, the ethical review and clinical trial registration are finalized. Participant recruitment is progressing well, with 100 individuals enrolled so far. Data collection is ongoing, and formal data analysis has not yet been initiated ().

## Discussion

Low back pain is the most prevalent chronic condition among the working population and a leading cause of disability and absenteeism. More than 80% of people will experience back pain at some point in their lifetime [35]. Studies have shown that low back pain can significantly impact daily activities, work performance, and sexual function [11,36]. Patients with low back pain frequently experience degeneration in the structure of the lumbar paravertebral muscles due to protective postures or muscle disuse due to the pain, along with increased inflammatory mediators.

Studies have demonstrated aerobic metabolic adaptations during trunk stretching in patients with chronic low back pain, who exhibit reduced mechanical efficiency, muscle endurance, and strength compared to healthy individuals [37]. The current study used a muscle hardness tester to monitor changes in muscle tension in patients with NSLBP before and after Tuina treatment combined with medicated oil. Current evidence suggests a correlation between trunk muscle strength, lumbar spine mobility, and the risk of low back pain. It has been noted that decreasing muscle tension in the waist can effectively alleviate low back pain symptoms, enhance joint mobility, and improve daily functioning [38]. Some studies have found that massage therapy can effectively relax the muscles and intervertebral joints surrounding the lumbar spine, correct spinal dysfunction, alter spinal biomechanics, and promote recovery from lower back pain [23,39-41]. In addition to patient-reported pain and functional outcomes, the current study incorporates infrared thermography and muscle hardness as objective secondary measures to enhance the clinical interpretability of treatment effects. In NSLBP, abnormal local skin temperature and increased paraspinal muscle stiffness are commonly associated with impaired microcirculation, persistent muscle spasm, and heightened nociceptive sensitivity. Improvements in skin temperature following intervention are interpreted as indicators of enhanced local blood flow and metabolic activity, which are physiologically linked to pain modulation and tissue recovery [42,43]. Similarly, reductions in muscle hardness reflect decreased muscle hypertonicity and improved elasticity, changes that are closely related to enhanced lumbar mobility and functional performance [37,44]. Although the measures of scales are not direct clinical end points, they provide objective evidence of underlying physiological recovery that supports and complements clinically meaningful improvements observed in pain intensity (VAS) and functional status (JOA). The multidimensional outcome framework helps bridge the gap between subjective symptom relief and objective biological change, thereby strengthening the clinical relevance and mechanistic interpretation of TNM in the management of NSLBP.

Infrared thermal imaging is widely used in cardiovascular medicine, sports medicine, ophthalmology, urology, and orthopedics [45-49] to visualize and observe temperature changes in skin, subcutaneous tissues, and blood vessels. The FLIR ONE Pro is a new type of infrared camera that is more convenient, affordable, and portable. A study has shown that people with lower back pain experience a significant increase in local infrared temperature after massage therapy compared to before treatment [50]. However, no research uses infrared thermography to evaluate local microcirculation changes in patients with NSLBP treated with TNM. Studies indicate significant differences between infrared thermal imaging methods in the average and highest skin temperature parameter values, reminding us that it is crucial to unify equipment and parameters for the observation results in the next step of conducting multicenter clinical studies.

The American College of Physicians clinical practice guidelines recommend using nonpharmacological therapies to treat NSLBP [51]. Tuina has unique advantages as one of the traditional external treatments of TCM. Several studies have demonstrated the benefits of manual therapy for both acute and chronic lower back pain. It can relieve muscle spasms, promote local metabolism, absorb damaged tissue, and eliminate edema [52]. A systematic analysis shows moderate-quality evidence that spinal manipulative therapy has similar effects on short-term pain relief as other recommended treatments. Medicated oil is a kind of Tuina ointment preparation widely used in clinical practice. It is mainly used for bruises and various trauma and pain syndromes. Promoting blood circulation and relieving pain are common indications of preparations [53].

A study has found that using local rose essential oil has a therapeutic effect on women with pregnancy-related lower back pain. After 4 weeks of treatment, compared with the base oil group and the no-intervention group, the rose essential oil group showed a significant reduction in pain intensity and improvement in daily activities [38]. Cayenne, devil’s claw, willow bark, comfrey, Brazilian arnica, and lavender essential oil have limited evidence of efficacy but suggest a possible benefit [54,55].

Some implementation difficulties need to be addressed in the research plan; we have designed corresponding measures to address these issues. First, to objectively evaluate the therapeutic effect more accurately, we used a muscle tension tester and an infrared thermal imaging device to assess the efficacy of TNM in treating patients with NSLBP. Under the premise of applying a unified standard Tuina method, we compared the effects of medicated oil and water on the experiment. Then, to promote and use the standardized technology of NSLBP clinical massage, a preliminary project launch meeting will be held to discuss the treatment plan with multiple experts in the field, simplifying the process to make it highly operable. To ensure the accuracy of the experimental intervention, all personnel involved in the project, including those implementing the treatment and evaluating the results, have received separate training. Each research group member is clear about their responsibilities, cooperates effectively, and completes the project on time. We standardized the appearance, specifications, and odor of the bottles to eliminate the placebo effect and compared them with water and activating oil, which will benefit clinical promotion and application.

The results are evaluated in 2 stages: the effects of lower back pain, motor function, muscle hardness, and temperature on 2 groups of patients with NSLBP were examined during treatment. To observe changes in pain and lumbar function during the follow-up period to evaluate the dependence of pain and movement disorders on muscle function and temperature.

A limitation of this study is that longitudinal correlation parameters could not be incorporated into the initial sample size calculation due to the lack of multiple time points pilot data. However, empirical intraclass correlation coefficients derived from the completed trial will be reported to inform the design of future multicenter randomized studies.

Nevertheless, we acknowledge that partial unblinding of therapists or participants cannot be entirely excluded, which represents a standard limitation in manual therapy trials. The assessor’s blinding, standardized intervention delivery, and inclusion of objective outcome measures (infrared thermography and muscle tension testing) are emphasized to mitigate potential bias due to expectations.

The combination of Tuina and medicated oil therapy for NSLBP is easy to operate. If time and space are limited, medicated oil can be used alone to apply to the waist. This study is a suitable option for patients with NSLBP who opt for conservative treatment, aiming to investigate the effects of Tuina Gao Mo on pain, functional activity, and emotional state. If this study’s findings are compelling, this approach can be further clinically promoted and applied.

## Abbreviations

AE: adverse event
CRF: case report form
FEMO: Flying Eagle Wood Lok Medicated Oil
JOA: Japanese Orthopaedic Association scores
NSLBP: nonspecific low back pain
TCM: traditional Chinese medicine
TNO: Tuina combined medicated oil
TNW: Tuina combined water
VAS: visual analog scale
